# Mesenchymal Stromal Cells Based Therapy in Systemic Sclerosis: Rational and Challenges

**DOI:** 10.3389/fimmu.2018.02013

**Published:** 2018-09-13

**Authors:** Juliette Peltzer, Marc Aletti, Nadira Frescaline, Elodie Busson, Jean-Jacques Lataillade, Christophe Martinaud

**Affiliations:** ^1^Unité de Thérapie tissulaire et traumatologie de guerre, Institut de Recherche Biomédicale des Armées, Clamart, France; ^2^Service de Médecine Interne, Hôpital d'Instruction des Armées Percy, Clamart, France; ^3^UMR7648 Laboratoire de physique des plasmas, École Polytechnique, Palaiseau, France; ^4^Unité de Médicaments de Thérapie Innovante, Centre de Transfusion Sanguine des Armées, Clamart, France

**Keywords:** cell therapy, good manufacturing procedures, immunomodulation, mesenchymal stromal cells, systemic sclerosis

## Abstract

Systemic Sclerosis (SSc) is a rare chronic disease, related to autoimmune connective tissue diseases such as Systemic Lupus Erythematosus and Sjögren's Syndrome. Although its clinical heterogeneity, main features of the disease are: extensive tissue fibrosis with increase matrix deposition in skin and internal organ, microvascular alterations and activation of the immune system with autoantibodies against various cellular antigens. In the diffuse cutaneous scleroderma subtype, the disease is rapidly progressive with a poor prognosis, leading to failure of almost any internal organ, especially lung which is the leading cause of death. Primary trigger is unknown but may involve an immune process against mesenchymal cells in a genetically receptive host. Pathophysiology reveals a pivotal role of fibrosis and inflammation alterations implicating different cell subtypes, cytokines and growth factors, autoantibodies and reactive oxygen species. Despite improvement, the overall survival of SSc patients is still lower than that of other inflammatory diseases. Recommended drugs are agents capable of modulating fibrotic and inflammatory pathways. Cellular therapy has recently emerged as a credible option. Besides autologous hematopoietic stem cell transplantation which demonstrated remarkable improvement, mesenchymal stromal cells (MSCs) represent promising therapeutic candidates. Indeed, these cells possess anti-inflammatory, antiproliferative, antifibrotic, and immunomodulary properties especially by secreting a large panel of bioactive molecules, addressing the most important key points of the SSc. In addition, these cells are very sensitive to their environment and are able to modulate their activity according to the pathophysiological context in which they are located. Autologous or allogeneic MSCs from various sources have been tested in many trials in different auto-immune diseases such as multiple sclerosis, Crohn's disease or systemic lupus erythematosus. They are characterized by a broad availability and no or low acute toxicity. However, few randomized prospective clinical trials were published and their production under ATMP regulatory procedures is complex and time-consuming. Many aspects have still to be addressed to ascertain their potential as well as the potential of their derived products in the management of SSc, probably in association with other therapies.

## Introduction

The word “scleroderma” originates from two Greek words: “sclero” meaning hard, and “derma” meaning skin due to skin hardening being one of the most visible manifestations of the disease. Scleroderma (also known as systemic sclerosis or SSc) is a multisystem connective tissue disease classified as autoimmune due to the presence of autoantibodies detected in nearly all patients (1, 2). SSc is a rheumatic disease with heterogeneous clinical manifestations and variable course, but, a characteristic clinical picture includes skin fibrosis, Raynaud's phenomenon, vascular disturbances, joint pain, digital ulcers, and telangiectasia. Internal organ changes such as gastrointestinal tract, lung, heart, and kidney dysfunction precede or simultaneously occur with cutaneous changes (1). Functional impairment of the hands caused by sclerodactily, flexion contractures of the fingers and stiff hands have a significant impact on quality of life of those overcome by SSc. This was the case with a prolific painter Paul Klee who after the diagnosis was forced to discontinue playing violin and his ability to paint was greatly restricted (3). Fatal health complications associated with SSc took Paul Klee's life in 1940. Since that time the understanding of SSc pathophysiology has evolved significantly. Many promising targets aimed at alleviating patient suffering and halting progression of painful and often lethal outcomes of this disease continue to be identified. Immunomodulation and stem cell-based therapy are emerging as an effective strategy in the treatment of SSc patients with poor prognosis (4). Pro-angiogenic, anti-inflammatory, and immunomodulatory properties of multipotent mesenchymal stromal cells (MSCs) make them ideal candidates for targeted cell-based therapy capable of restoration of immune functionality or in other words, immune “reboot” in autoimmune diseases. In this review we discuss therapeutic potential and rationale for application of mesenchymal stromal cells in the treatment of SSc.

## Clinical aspects

### Epidemiology

Although epidemiological data related to the incidence and prevalence of SSc vary depending on geographical region and period of observation (5), worldwide prevalence is estimated to range from 50 to 300 cases per 1 million persons, whereas the incidence is predicted to range from 2.3 to 22.8 cases per 1 million persons per year (1). SSc prevalence and incidence estimates are consistently higher in the USA and Australia (prevalence is 276–443 per million; incidence is 14–21 per million per year) compared to Europe and Japan (prevalence is <150 per million; incidence is <10 per million per year). The highest prevalence of SSc is amongst a Choctaw Native American group in Oklahoma with an estimated prevalence of 660 cases per million suggesting that genetic factors and predisposition of certain ethnic and racial backgrounds may play a role in SSc etiology (5). There exists a gender disparity when it comes to SSc prevalence—a common factor shared by more than 70 other autoimmune disorders. When it comes to SSc disease expression women have a much higher susceptibility than men with a ratio ranging from 3:1 to 14:1 (1).

### Clinical features

There are several subsets of SSc. According to LeRoy and colleagues (6), SSc can clinically be differentiate into 3 main phenotypes: (i) limited cutaneous systemic sclerosis (lcSSc), (ii) diffuse cutaneous systemic sclerosis (dcSSc), and (iii) SSc sine scleroderma. In lcSSc, skin involvement is limited to areas distal to the knees and elbows, and often just to the wrist and ankles. Skin changes on the face and neck may also be evident. There is typically a long antecedent history of Raynaud's phenomenon, often severe and associated with recurrent digital ulceration. Other manifestations include oesophageal dysmotility, gastro-oesophageal reflux, cutaneous telangiectasia, which is generally seen on the palms and around the mouth, and subcutaneous calcinosis. The term lcSSc is preferred to CREST syndrome as it does not ignore the important internal organ manifestations of mid-gut disease (small bowel bacterial overgrowth), pulmonary fibrosis and pulmonary arterial hypertension. Anticentromere antibodies are the hallmark antibodies in this condition, although other antibodies may also be present. The formal classification of dcSSc is determined by the presence of skin sclerosis proximal to the knees and elbows, and usually affecting the trunk. The classical presentation is an abrupt onset of inflammatory change in the skin and other structures. Pain and swelling of the extremities often occur and expansion of tissues around the wrist often results in bilateral carpal tunnel syndrome. Tendon friction rubs can be felt across joints. Affected skin is often intensely pruritic with a loss of specialized skin structures leading to changes in perspiration and hair growth. Raynaud's phenomenon develops simultaneously with other features or once the disease is established. Oesophageal involvement is almost universal and severe internal organ complications tend to occur earlier in the course of disease compared with lcSSc. Lung fibrosis or hypertensive renal crises are relatively frequent, and some specific antibodies can be predictive of these. The autoantibodies classically associated with hypertensive scleroderma renal crisis are Scl-70, antifibrillarin, and anti-RNA polymerases I and III. In addition to these are the less-commonly occurring anti-Pm-Scl and anti-nRNP autoantibodies, which have also been reported to occur in patients with connective tissue diseases other than SSc. The natural history of this condition is heterogeneous and skin sclerosis can remit after several years despite progression of internal organ disease. Finally, SSc sine scleroderma is observed in patients with typical vascular features and serological changes associated with SSc together with visceral complications, such as lung fibrosis, hypertensive renal crisis or severe bowel involvement, but without any evidence of skin fibrosis. This clinical profile is termed SSc sine scleroderma and probably accounts for <1% of cases, although it may well be underdiagnosed (7).

### Outcome and prognostic factor

SSc is associated with high morbidity and mortality. Five and ten-years survival rates, although improving, are in the order of 68 and 50% respectively (8). This is highlighted in a recent study showing an age and sex adjusted standardized mortality ratio of 4.06 for newly diagnosed SSc patients, with 22.4 and 26.0 years of loss of life in women and men, respectively (9). Disease-related causes, in particular pulmonary fibrosis, pulmonary arterial hypertension, cardiac complications and renal crisis, accounted for the majority of deaths in SSc (10). Malignancy, sepsis, cerebrovascular disease, and ischemic heart disease are the most common non–SSc-related causes of death. Predictors of early mortality included male gender, older age at disease onset, diffuse disease subtype, pulmonary arterial hypertension, and renal crisis (9).

### Disease classification

The diagnosis of SSc is based on recognition of specific features and physician judgment. In 2013, American College of Rheumatology (ACR)–European League Against Rheumatism (EULAR) established a new classification criteria for SSc incorporating important elements (proximal scleroderma, sclerodactyly, digital pits, pulmonary fibrosis, Raynaud's phenomenon, and scleroderma specific autoantibodies) (11). The new criteria placed more emphasis on the vasculopathic manifestations of the disease and accounted for other clinical features including puffy fingers—another pivotal sign of SSc. The new criteria is more specific, especially when it comes to identifying early stages, mild or limited form of the disease. Classification criteria is used to identify homogenous groups of patients for inclusion into studies and can be used to identify the disease stage and progression. Patients with a total score of ≥9 are classified as having SSc.

## Rational of MSC-based therapy of SSc

### Physiopathology

SSc is characterized by a triad of vascular damage, aberrant inflammatory response and tissue fibrosis (12). Fibrosis gradually replaces healthy tissue and ultimately disrupts the architecture of the affected area causing debilitating symptoms. In fibrotic tissue, normal architecture is replaced with collagen rich, largely acellular, stiff connective tissue resulting in loss of functional integrity consequently leading to severe dysfunction and, in some cases, failure of vital internal organs including the heart and lungs–fatal complications of SSc (13). Pathogenesis of SS-associated fibrosis has been the subject of extensive research, and, although gaps in knowledge still exist, common features of fibrosis are becoming clear regardless of the organ or tissue it affects. Common features of SSc fibrosis include: (i) increased presence and persistence of differentiated fibroblasts (myofibroblasts); (ii) excessive deposition of extracellular matrix components, caused by overproduction of collagen and other glycoproteins and (iii) increased tissue contraction. SSc is characterized by vascular injury which is an early event preceded by fibrosis. A common consensus is that, a primary vascular and immune event cause fibroblast activation, which further activates innate immune signaling resulting in a vicious cycle of fibrogenesis (12, 13). Vascular injury and reduced blood supply lead to progressive tissue hypoxia, which further stimulates collagen synthesis and contributes to the progression of fibrosis in SSc (12). Activated inflammatory cells secrete cytokines causing fibroblast differentiation into myofibroblasts, which produce excessive collagen, contract and induce pathological changes in the connective tissue. Chronic inflammation and persistent presence of pro-inflammatory cytokines and growth factors further drive excessive accumulation of extracellular matrix (13).

Biological specimen of skin, lungs (including bronchoalveolar lavage fluid), and other organs affected by SSc, serve as study tools, which assist in unrevealing possible mechanisms involved in SSc pathogenesis. It has been suggested that an initial event in the form of genetic mutation or environmental trigger, induces autoimmunity and autoantibody production, which in turn activates innate immune cells (such as resident macrophages) and the secretion of innate immune cytokines leading to chronic inflammation (13). At the same time, adaptive immune response is activated and T_H_1 cells are mobilized. T_H_1 cells are known to be responsible for the secretion of inflammatory cytokines and growth factors, whereas T_H_2 cells are predominantly profibrotic (12). Hence, it has been concluded that autoimmunity and inflammation activate fibroblasts and result in pathological fibrogenesis (13). This hypothesis was formed based on histological and molecular analysis of SSc specimen which demonstrate the presence of mononuclear-cell infiltrate (1) including bone marrow-derived CD4^+^ T cells, macrophages, activated B cells, dendritic, mast cells, and other markers of inflammation (14). Interestingly, an overwhelming presence of CD4^+^ T cells, type 2 T helper (T_H_2) cells known to secrete IL-4 and IL-13 and to a lesser extent T_H_1 cells, which primarily secrete anti-fibrotic interferon γ (IFNγ) was detected as part of the cellular infiltrate in SSc biopsy samples of skin (12). Similarly, macrophages have been identified as important players of SSc-associated inflammation. M2–also known as alternatively activated macrophages were detected in skin and lung biopsies, whereas soluble levels of CD163 (known M2 marker) in sera of patients with SSc were significantly elevated compared to controls further suggesting a role for this immune cell in disease pathogenesis (12).

### Overview of current therapeutic approaches

Chronic inflammation and autoimmunity are cardinal pathogenic events associated with SSc and hence, logically, therapeutic targeting of either of these processes is likely to be beneficial. Although current therapeutic approaches include general immunosuppression and complication-specific therapies, immunomodulatory regimens with myelosuppression or myeloablation followed by autologous haematopoietic stem cell (HSCs) transplantation (4, 15–17) have been evaluated as a therapeutic strategy in systemic sclerosis (Table 1).

**Table 1:** The updated EULAR recommendations for treatment of systemic sclerosis, according to the organ involvment, including strength of the recommendation.

**Organ involvement**	**Recommendation**	**Strength of recommendation**
I. SSc-RP	To reduce the frequency and severity of SSc-RP attacks: - First line therapy: dihydropyridine-type calcium antagonists (nifedipine) or PDE-5 inhibitors - Second line therapy: intraveinous prostanoids (iloprost) - Other: fluoxetine	A A C
II. Digital ulcers on patients with SSC	In treatment of digital ulcers in patient with SSc: - intravenous iloprost - PDE-5 inhibitors To prevent development of new digital ulcers in SSc: - PDE-5 inhibitorTo reduce the number of new digital ulcers in SSc, especially in patients with multiple digital ulcers despite use of calcium channel blockers, PDE-5 inhibitors or iloprost therapy: - Bosentan	A A A
III. SSC-PAH	To treat SSc-related PAH and CTD-PA: - ERA (ambrisentan, bosentan and macitentan) - PDE-5 inhibitors (sildénafil, tadalafil) - Riociguat - Intravenous epoprostenol (severe SSC-PAH with class III and IV dyspnea) - Prostacyclin analogs	B A B
IV. Skin and lung disease	To treat skin manifestations of early diffuse SSc: - Mehtotrexate Treatment of SSc-ILD, in particular for patients with SSc with progressive ILD: - cyclophosphamide Treatment of selected patients with rapidly progressive SS and risk of organ failure: - HSCT should be considered	A A A
V. SRC	- ACE inhibitors	C
VI. SSc-related gastro intestinal disease	To treat SSc-related GERD and prévention of oesophageal ulcers and stricture - PPI To manage SSc-related symptomatic motility disturbance (dysphagia, GERD, early satiety, bloating, pseudo-obstruction…) - Prokinetic drugs To treat symptomatic small intestine bacterial overgrowth: - intermittent or rotating antibiotics	B C D

CTD, connective tissue disease; ERA, endothelin receptor antagonists; GERD, gastro-oesophageal reflux disease; HSCT, haematopoietic stem cell transplantation; PAH, pulmonary arterial hypertension; PDE-5, phosphodiesterase type 5; PPI, proton pump inhibitor; RCTs, randomized contrpolled trials; SRC, scleroderma rénal crisis; SSC, systemic sclerosis; SSC-RP, Raynaud's phenomenon in patients with SSC; ILD: intersitial lung disease

The rationale behind autologous stem cell therapy is that after profound depletion of immune cells, including autoreactive T and B cells, a new and naive immune system originating from the stem cell graft will re-establish immune tolerance. Apart from the presence of inflammatory infiltrate and markers of chronic inflammation in diseased specimen another observation supports the rational for profound immunomodulation as a therapeutic approach in SSc. This includes clinical overlap of SSc with other autoimmune rheumatic diseases where immunomodulation in the form of stem cell therapy reset immunological clock, and was associated with sustained disease remission (18) further adding to the evidence and supporting the concept of cell-based targeted therapies in the treatment of SSc.

To date, a number of stem cell transplantation studies were shown to be effective in international multicenter clinical trials demonstrating sustained improved clinical effects especially when it comes to late stage disease diffused cutaneous SSc (dcSSc) (15, 17, 19). Autologous stem cell therapy offers the patient an opportunity to “reboot” the immune system and is currently the only curative measure that is able to induce sustained clinical improvement and long-term drug-free remission compared to short-lived (and often ineffective) results of “debulking of inflammation” with cytotoxic and immunosuppressive regimens (17, 20). Mesenchymal Stromal Cells (MSCs) with their immunomodulatory and anti-inflammatory properties may provide an alternative to HSC with a potential to provide long-term benefits in patients with scleroderma (18, 21). Further along we will discuss biological properties of MSCs and elaborate of their potential use for cell-based therapy in the context of SSc.

MSCs are well known as very well tolerated and hence, it is hoped that the use of MSC-based therapy may be used as an adjuvant treatment or may indeed replace immunosuppressive drug therapy. The feasibility of cell therapy, regardless of the cell origin and mode of administration, in adult patients has already been demonstrated and MSCs are considered safe. Indeed, to date, reviews of reported adverse events in clinical trials failed to demonstrate side effects, especially when it comes to toxicity, infection, death or tumorigenicity (22, 23).

### Mesenchymal stromal cells: origin and properties

The beginnings of cell therapy using so-called MSCs go back several decades. However, we are still far from fully understanding the complex nature of these cells. Early MSC characterization studies appeared at the time when Bone Marrow (BM) transplantation was born. The first human BM transplantation took place in 1957, which resulted in hematopoietic reconstitution following an accidental irradiation (24). Subsequently, the first heterotopic BM transplantation was able to highlight the osteogenic potential of medullary cells (24). During the 1960s, the characterization of various cellular constituents of BM gradually begun. Alexander Friedenstein discovered the existence of fibroblastic multipotent progenitors isolated by culture plastic adhesion from BM but also from spleen (25). He also demonstrated the ability of these culture-isolated fibroblast cells to recreate a hematopoietic environment *in vivo* after heterotopic grafting (26). These founding experiments also provided the first clues to the existence of a memory of the original tissue. These cells of similar appearance favoring lymphopoiesis or myelopoiesis according to their medullary or splenic origin. Arnold Caplan later introduced the term mesenchymal stem cell in the early 1990s and showed that these cells were able to generate cartilage, tendons and muscle *in vitro* (27). Finally, in the 2000s, the lack of convincing data to assert the stemness of MSCs, as defined by Loeffer and Potten in 1990 (28), caused the International Society for Cellular Therapy (ISCT) to make an amendment to existing terminology, hence thereafter these cells were termed Mesenchymal Stromal Cells. This allowed to keep the same acronym and to highlight their trophic capacities (29). More recently, it has been shown that MSCs can be isolated from different mesodermal support tissues as well as perinatal tissues (30).

The *in vitro* differentiation capabilities of MSCs were the first to attract the attention of clinicians. This initially led to suggest their use in repair of musculoskeletal defects (27, 31). Gnecchi's team in 2005 used MSCs after myocardial infarction. A significant reduction in the size of infarcted area and apoptotic cell index were recorded as early as 72 h after MSCs injection. It was suggested that as the myocardium assessment was carried out shortly after the treatment with MSCs the likelihood of cardiomyogenic differentiation of MSCs is unlikely. It was then hypothesized that this protective action was related to the secretion of paracrine factors by MSCs. To test this hypothesis, the group produced conditioned media from MSCs cultures and injected this media into occluded coronary arteries of rats. Beneficial effects of cardioprotection have been observed with the use of conditioned media (32, 33).

Other studies based on BM transplantation trials have been performed to treat hematopoietic disorders. Indeed, MSCs derived from the medullary microenvironment participate in the regulation of self-renewal and differentiation of HSCs. In the 2000s, injection of autologous MSCs after myeloaplasia and autologous HSCs transplantation was shown to lead to an earlier resolution of aplasia (34). Moreover, it has been shown by several teams that the co-graft of MSCs and HSCs from the same donor allowed for better engraftment of HSCs while decreasing the risk of graft-vs.-host reaction (GvHD) (35, 36). Finally, the study carried out by the team of Le Blanc et al. on patients suffering from GVHD has shown that injections of haploidentic MSCs could have an immunosuppressive effect *in vivo* (37). All these studies led to the idea that the efficacy of MSCs was probably more related to the secretion of factors regulating endogenous cell activity, than by differentiation to replace damaged cells.

There exist multiple modes of communication used by MSCs. These include secretion of a wide range of bioactive molecules (cytokines/chemokines/growth factors), direct cellular communication through the expression of different membrane markers, mitochondrial transfers and production of extracellular vesicles (EVs) containing proteins, mRNA, miRNA together with mitochondrial fragments. EVs is a collective term for different types of membrane-surrounded structures with overlapping composition, density, and sizes (ranging from 20 to 1,000 nm in diameter), including exosomes, ectosomes, microvesicle particles and apoptotic bodies in accordance with the recommendations of the International Society for Extracellular Vesicles (ISEV) (38). These EVs are released from most types of cells and serving as a means of communication. EVs released from different cells have been implicated in a host of normal cell functions including immune modulation, tissue repair, reproduction, or cancer progression (39). Moreover, EVs released into body fluids have been shown to serve as biomarkers for disease states. Their content, their mode of release or uptake vary between cell types and have also been shown to play a role in the elimination of unwanted cellular components or drugs (39). Recent studies demonstrated that EVs represent a powerful component of the MSCs secretome which could play an important role in tissue regeneration (38).

Finally, the identification of factors that can influence the effectiveness of MSCs has led to a better understanding of interactions between MSCs and their “targets.” This also highlighted their great sensitivity to their environment. The modulation of the environment can be chemical, physical or dependent on matrix support and cellular interactions. Therefore, it is important to characterize pathophysiological context in which MSC could be useful and optimize their therapeutic benefit by modulating cellular culture environment before their administration. This forms the basis of cellular “priming” concept. The concept of priming has a twofold aim, firstly, to prepare MSCs to the environment, in which they will be administrated and, secondly, to modulate their behavior to counteract or promote a desired physiological response. Current research aims to understand “innate” and “acquired” components that influence MSCs activity. It must be kept in mind that the concept of cell memory, which enables a cell to “remember” it's tissue or organ origin is as essential as the priming conditions applied before the cell is delivered to the target site.

#### MSCs and immune system

SSc is an autoimmune disease with altered cellular immunity, including T and B lymphocyte functional disturbances (40). Moreover, recent data showed an aberrant dendritic cell function (41) and that although the number of natural Tregs is increased during SSs, their ability to suppress CD4+ effector T is impaired (42). Autologous HSCT for severe autoimmune disease has demonstrated remarkable improvement in many patients. Even if the emergence of new biologic agents reduced the need of cellular therapy for several pathologies such as rheumatoid arthritis and multiple sclerosis, it still remains an option for SSs (20). However, on 35 patients receiving allogeneic HSCT for autoimmune disease 50% showed remission (43). In addition, this strategy also involves the risk of developing GvHD.

MSC therapy in humans in the context of bone marrow graft enhancement (34) but also in the context of acute GvHD (37) was already described as a potentially effective therapy. Following these pioneer studies, many authors highlighted the capacity of MSCs to inhibit the mixed lymphocyte reaction independent of HLA restriction in 2003 and showing anti-proliferative, anti-inflammatory and immunomodulatory properties of MSCs on all actors of the immune system (20). Indeed, it was described that MSCs could favor monocyte polarization to anti-inflammatory M2 macrophages which increase the production of IL-10 and decrease the production TNF-alpha and IL-12 (44). However, MSCs are very sensitive to their environment and we already demonstrated *in vitro* that they can be reversed from having a suppressive to supportive phenotype when exposed with defective immune cells. We further showed that this immune activating effect may be due to MSC pre-stimulation (45). Therefore, before any clinical application, the plasticity of MSCs should be carefully considered and explored in the pathophysiological context in which they will be used.

Only few studies have investigated the immunoregulatory activity of MSCs in the context of SSs. Recent report indicated that SSs-MSCs showed impaired proliferation, differentiation, secretion of cytokines and immune modulation (21). However, two *in vitro* studies have reported that SSc-MSCs, although senescent, might display the same immunosuppressive properties *in vitro* as their healthy counterparts. These observations were realized by using co-culture models between MSCs and peripheral blood mononuclear cells (PBMCs) (46). Moreover, SSc-MSCs could favor Tregs population (46).

Recent studies showed that systemic infusion of BM-MSCs induced transient T cell apoptosis via the FAS ligand (FASL)-dependent FAS pathway. Then, the apoptotic T cells triggered macrophages to produce high levels of TGF-β, which in turn led to the upregulation of CD4+CD25+Foxp3+ regulatory T cells and could ameliorate the disease phenotype in fibrillin-1 mutated SSc mice. This mechanism is not observed after the administration of FASL-/- BM-MSCs. Moreover, they demonstrated that BM-MSCs recruited T-cells for FASL-mediated apoptosis through the secretion of Monocyte Chemotactic Protein 1 (MCP-1) (47). Another study also demonstrates that allogeneic MSCs could attenuate a mice model of cutaneous sclerodermatous GVHD by selectively blocking immune cell migration and down-regulating chemokines and chemokine receptors (48).

#### MSCs and vascular system

SSc is characterized by a widespread vasculopathy, defective angiogenesis and progressive fibrosis of the skin and internal organs (49). This vasculopathy is related to endothelial cell (EC) dysfunction which interferes with the cell survival (activation and apoptosis), angiogenesis and vasculogenesis and also by their interactions with various other cells (50, 51). Indeed, despite marked tissue hypoxia, there is no evidence of compensatory angiogenesis in SSc (52). In the process of vasculogenesis, endothelial progenitor cells (EPCs) are mobilized from the BM to the site of neovascularization and differentiate into mature endothelial cells. In SSc, reduced number of EPCs has been detected (52). It was thought that MSCs are an alternative source of EPCs because they display some features of mature endothelial cells, such as the expression of von Willebrand factor (vWF), vascular endothelial growth factor receptor 1 (VEGFR-1), VEGFR-2, VE-cadherin, and vascular cell adhesion molecule 1 (VCAM-1) (52). It was shown that SSs MSCs expressed less VEGFR-2, CXCR4, VEGFR-2/CXCR4 cells than MSCs from healthy controls and early senescence was detected (52).

Furthermore, it has already been described that MSCs could play a crucial role in the modulation of angiogenesis in several models like hindlimb ischemia (53, 54). They were able to produce cytokines and growth factors which could protect endothelial cells from apoptosis and to promote angiogenesis (55). Therefore, several authors investigated the possible paracrine therapeutic role of MSCs in the pathogenesis of SSc. Guidicci et al. showed that BM-derived MSCs from patients suffering from early severe and rapidly progressive diffuse SSc (SSc-MSCs) overexpress bioactive mediators and pro-angiogenic growth factors in contrast with BM-MSCs issued from healthy donors. SSc-MSCs seems to be influenced by the local microenvironment (stimulation by vascular endothelial growth factor (VEGF), transforming growth factor β (TGFβ) or stromal cell-derived factor-1 (SDF-1) and upregulate the release of these factors in response to these exogenous stimuli. They also showed that SSc-MSC-conditioned medium had a greater pro-angiogenic effect on dermal microvascular endothelial cell (MVECs) *in vitro* than healthy MSCs donors (49).

Cipriani et al. also showed that environmental cues associated with SSc seem to induce an upregulation of α-SMA and SM22α gene expression on perivascular BM-MSCs and a downregulation of their proliferative activity. Moreover, by using BM-MSCs and healthy human MVECs coculture system the group observed that BM-MSCs isolated from patients with SSs (SSs BM-MSC) like BM-MSCs from healthy patients (H BM-MSC) were able to improve endothelial cell tube formation in stressed condition. Finally, it was shown that co-culture of SSc BM-MSC with healthy MVECs reverts the expression of contractile gene apparatus (α-SMA and SM22α). The authors concluded that SSc BM-MSCs display a more mature and myofibroblast-like phenotype and that their coculture with endothelial cells re-programs these cells toward a pro-angiogenic behavior (56).

In a case report in 2010, the team of Guidicci used intravenous infusion of expanded autologous MSCs in a patient with critical limb ischemia due to SSc. Angiography showed that MSCs enhanced revascularization of patient's extremities. Moreover, histological skin analysis revealed cell clusters with tube-like structures and an increase expression of angiogenic factors. These data suggested that MSCs could promote vascular network recovery in case of severe peripheral vascular disease in a patient with SSc (49).

#### MSCs and fibrosis

Microvascular damage that causes tissue hypoxia is pivotal in the pathogenesis of SSc and it preceding fibrosis. Fibrosis may be considered as the main characteristic of SSc and affecting not only the skin but also all internal organs (57). Fibrosis is characterized by an excessive production of collagen and thickening of the skin and connective tissue caused by a fibroblast dysfunction (58). A large number of soluble paracrine mediators have been implicated in fibrosis and notably, the transforming growth factor (TGF)- β signaling pathway which enhance the pro-fibrogenic cellular programs (59). TGF-β, which is mainly produced by fibroblasts and T helper type 2 lymphocytes, is the major cytokine involved in collagen production leading to fibrosis. TGF-β is regulated by TGF-β receptors (TBRI and TBRII) expression level (60). Moreover, activated fibroblasts are the key effector cells in SSc. Platelet-derived growth factor (PDGF), a potent mitogen for cells of mesenchymal origin, has been implicated in the activation of fibroblasts in SSc. Therefore, inhibition of PDGF signaling could be an attractive therapeutic approach for SSc (61). It had already been demonstrated that BM-MSCs could have a benefit on fibrosis development in different organs including: lung (62), kidney (63), heart (32) or skin (64).

**Figure 1:**
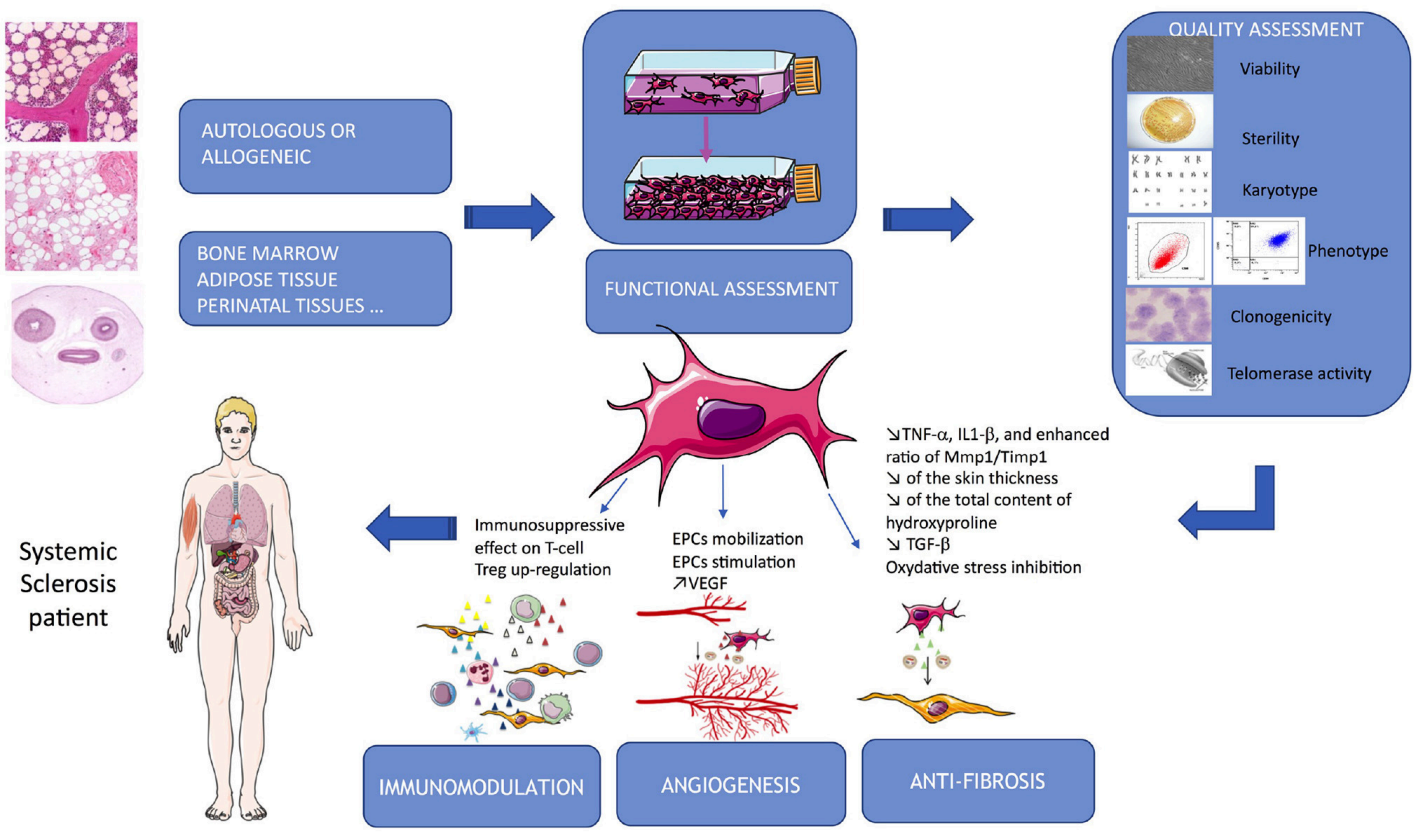
Summarize of MSCs-based therapy in SSc. MSCs are issued from different autologous or allogeneic tissues, their properties used in SSc target immunomodulation, angiogenesis and fibrosis. Quality assessment is a critical part of the process and required several tests allowing their delivery to the patients.

Administration of allogeneic vs. xenogeneic MSCs was evaluated in a mouse model of HOCl-induced diffuse SSc. Additionally, this team evaluate two distinct human MSCs sources: BM and adipose tissue. They showed that xenogeneic human BM-MSCs were as effective as allogeneic or syngeneic BM-MSCs. They decreased skin thickness, expression of Col1, Col3, α-SMA transcripts and collagen content in skin and lungs. Moreover, human Adipose-derived stem cells (ADSCs) were significantly more efficient to reduce skin fibrosis, which was related to a stronger reduction of TNF-α, IL1-β, and enhanced ratio of MMP1/TIMP1 in skin and lung tissues than BM-MSCs (65). Finally, they demonstrated that this anti-fibrotic effect was not associated with MSCs migration to injured skin or with their long-term survival. Indeed, Human MSCs were undetectable 7 days after infusion, whereas they observed a plateau of clinical fibrosis and a decrease of clinical symptoms 21 days after infusion. Finally, this team presumed that the progressive loss of effect might be due primarily to the early disappearance of cells rather than a putative progressive trans-differentiation of MSCs into endothelial cells (65). Moreover, in 2017 Chen et al. showed that in a murine model of bleomycin-induced cutaneous scleroderma, subcutaneous administration of ADSCs significantly attenuated bleomycin-induced dermal fibrosis, reduced skin thickness and total content of hydroxyproline. This team also explained that pathophysiology of SSc involves a complex interplay of inflammation, fibrosis, and vasculopathy. VEGF is a central regulatory factor for the formation of new vessels that controls angiogenesis and has protective effects in SSc patients. TGF-β1 is known to be a fibrosis stimulus factor in SSc. In their mouse model, the ADSCs treatment group showed significant lower levels of TGF-β1 and higher levels of VEGF than the control group (66). It is also already known that inhibiting oxidative stress protect against fibrosis. The thioredoxin (Trx) system is one of the principal intracellular redox systems and regulate its function. Jiang et al. investigated the therapeutic potential of BM-MSCs overexpressing Trx-1 to treat SSc skin after transplantation into a bleomycin-induced murine model exposed to 48 h of hypoxia. They showed that that Trx-1-overexpressing BM-MSCs inhibited hypoxia-induced apoptosis and fibrosis and also promoted the formation of tubular-like structures by endothelial progenitor cells. They suggested that the mechanism of inhibition of fibrosis could involve downregulation of TGF- β (51). These data also suggested that MSC priming could enhance their efficacy in SSc pathophysiology. Finally, another team showed that MSCs isolated from SSs patients showed significant increase in mRNA levels and membrane expression of TGF-β receptor types II (TBRII). Moreover, in response to TGF-β activation, SSs-MSCs showed a significant increase in collagen 1α synthesis and an upregulation of Smad-3 phosphorylation (60). These properties are summarized in Figure 1 and Table 2.

**Table 2:** Summarize of characteristic of SSc-MSCs and therapeutic targeting the main features of the disease.

	**Immune system**	**Vascular system**	**Fibrosis**	**References**
SSc-MSCs characteristics	↘ T-cell proliferation ↗ Of functionally CD4^+^CD25^bright^FoxP3^+^CD69^+^ cells ↗ Antioxidant capacity, notably the expression of SOD2 antioxidant gene **In contrast to other reports:** ↘ Capacities of proliferation ↘ Capacities of differentiation ↘ Secretion of cytokines ↘ Capacities of immune modulation	↗ Secretion of bioactive mediators and pro-angiogenic growth factors ↗ Tube formation by endothelial cells (enhanced under hypoxic conditions). SSc-MSCs coculture with endothelial cells → re-programs these cells toward a pro-angiogenic behavior	↗ mRNA protein expression of TGF-β receptor types II ↗ Collagen-1α synthesis ↗ Regulation of Smad-3 phosphorylation	(21) (46) (49) (56) (60) (67) (68) (69)
	**SSc-MSCs** = **literature discrepancy**	**SSc-MSCs** = **pro-angiogenic**	**SSc-MSCs** = **pro-fibrotic**	
Therapeutic MSCs	↗ CD3^+^ T cell apoptosis ↗ Treg population Blocking of immune cell migration and down-regulating chemokines and chemokine receptors	↗ revascularization ↗ number of clusters with tube-like structures ↗ expression of angiogenic factors ↗ level of VEGF	↘ Skin thickness ↘ Expression of Collagen-1α, Collagen-3, α -SMA transcripts and collagen content in skin and lungs ↘ skin fibrosis, which was related to: ↘ TNF- β1, IL1- β ↗Ratio of MMP1/TIMP1 in skin and lung tissues ↘ Levels of TGF-β1	(46, 47) (48) (49) (58) (65) (66)
	**MSCs** = **immunosuppressive**	**MSCs** = **pro-angiogenic**	**MSCs** = **anti-fibrotic**	

## MSC-based therapy in SSc

### Regulatory issues

In the EU, products containing living cells and/or products of gene therapy or tissue engineering efforts are regulated by the advanced therapy medicinal products (ATMP) regulatory path (70). The 2007 legislation replaced previous legislations, the EU 2001/83 and 2004/76, by creating an EU legislation regarding medicinal drugs and establishing procedures for authorization and monitoring. A Committee for Advanced Therapies (CAT) was created in charge of the classification of these products. ATMPs are defined as having properties for treating or preventing diseases in patients, or products that may be used in or administered to them with a view to restoring, correcting or modifying physiological functions by exerting principally an immunological, pharmacological, or metabolic action. The 2007 legislation also confirmed the innovative aspect of ATMPs and provided a legal definition of tissue engineered products and combined ATMPs. This directive, by defining the legal status of ATMP, determines its legal framework of manufacturing, development and market authorization. Gene therapy medicinal products and somatic cell therapy medicinal products were defined previously in the EU 2001/83 but the 2007 legislation precise that if a product can be related to the gene therapy definition as well as to the somatic cell therapy or tissue engineered products, then it will be considered as gene therapy product. In the same line, if a product meets both definition of tissue engineered product and somatic cell therapy, then it will be considered as tissue engineered product. Somatic cell therapy medicinal products are cells or tissues that have been subject to substantial manipulation, so that biological characteristics, physiological functions or structural properties relevant for the intended regeneration, repair or replacement are achieved. If the function in recipient and donor is different, the cells are no longer considered as cell therapy but ATMPs. Finally, combined advanced therapy medicinal product are advanced therapy medicinal product that incorporate one or more active implantable medical devices and viable cells or tissues, or non-viable cells or tissues which must be liable to act upon the human body with action that can be considered as primary to that of the devices referred to. At the end of the day, ATMPs are classified into 4 groups: gene therapy product, somatic cell therapy medicinal product, tissue engineered cell product and combined advanced therapy medicinal product. However, the scope of this Regulation is restricted to advanced therapy medicinal products which are intended to be placed on the market in EU and either prepared industrially or manufactured by a method involving an industrial process; Advanced therapy medicinal products which are prepared on a non-routine basis according to specific quality standards, and used within the same EU country in a hospital under the exclusive professional responsibility of a medical practitioner, in order to comply with an individual medical prescription for a custom-made product for an individual patient, are excluded from the scope of this Regulation. Although local regulations, these products are considered as ATMPs, there only difference is that they are manufactured for a single patient.

### Managing the variability

Due to their variability and plasticity, the nonclinical and clinical studies need to be carried out with well-defined and characterized MSCs. Clinical MSC-based batches have to be produced following a robust and standardized process and controlled for their quality and safety to ensure substantial and reproducible results. Several points may arise from each step of the production process of MSCs. Below main issues will be addressed.

#### Donor related variability: autologous vs. allogeneic

The question of using autologous or allogeneic MSCs in cell therapy trials often arises. Some groups suggested that MSCs survival may be increased with autologous than with allogeneic cells that should be easier rejected in immunocompetent patients. Even if several studies found that *in vitro* SSs-MSC immunomodulatory properties were similar to that of healthy donors (46, 67) other reported that SSs-MSCs exhibited impaired capacities of proliferation, differentiation, secretion of cytokines and immune modulation (21). Another study showed that BM-MSCs issued from SSs environment exhibit abnormal functional activities, such as increased expression of TGF-β and vascular endothelial growth factor (VEGF), and impairment of endothelial cell (60). Moreover, an impairment of MSCs endothelial cell differentiation capacities (52) and an overexpression of their secretion of proangiogenic factors was already described (49). Therefore, several data seemed to indicate that autologous approaches could be inappropriate because of functional alterations in MSCs from patients, whereas others described similar potential between healthy and SS-MSCs. This discrepancy in the literature indicated that this question has to be carefully considered for clinical use in SSc patients and highlight the need of further studies. Recent clinical trials using allogeneic MSCs illustrated that it could be an interesting option (71–73). Because of the age of the scleroderma patients, their poor health and thus the high probability that the MSCs proliferative power may be impacted, allogeneic donors should be preferred. But in this case of allogeneic use, French regulatory authorities now ask for the development of a Human Leucocyte Antigen (HLA) cross-match test between donor's MSCs and recipient's plasma, as MHC (Major Histocompatibility Complex) mismatch should be responsible for the lack of therapeutic effect.

Due to their heterogeneity within a population and their inter-donor variability, MSCs cultures for clinical use are hard to standardize (in case of autologous use). Our recent data of clinical BM-MSCs expansion (healthy individuals, *n* = 5) reveal a variability in bone-marrow richness of mononuclear cells between donors and a great variability in terms of cell proliferative potential. For instance, gap could be up to a factor 5.6 between the best donor and the worst (data not published). Previous studies suggest a decline of MSCs in bone-marrow with age when comparing groups with a bigger age difference. Caplan's team evaluated, thanks to CFU-F assays, that marrow of newborns, teens, 30, 50, and 80 years old donors would contain respectively about 0.01, 0.001, 0.0004, 0.00025, and 0.00005% of MSCs (74). In addition, exposure to environmental damage and stress over a long time seems to be responsible of negative effects on physical and biological properties of MSCs including proliferation, clonogenicity, differentiation, immunoregulation, paracrine secretion, life-span and senescence (75, 76). In consequence, donor's age is a criterion that should be taken into consideration when proceeding to a clinical trial.

#### MSCs sources

Despite the variety of source tissues and the common characteristics that all MSCs share (morphology, proliferation, clonogenicity, differentiation potential, plastic adherence, and a common surface marker profile), they can be more or less easily isolated according to the tissue. For example, ADSCs are described as cells more widely available (because of the high abundance of fat in the body), with a higher yield at isolation, easier to expand in culture and causing less donor-site morbidity than other sources. In addition, cells of different origin can differ by their paracrine activity potency and thus, by their functional properties. For example, Bortolotti et al. have shown that murine BM-MSCs had a better therapeutic potential than murine ADSCs in a preclinical model of critical limb ischemia (77). In this study, both cell types were able to reduce necrosis and inflammation, and to stimulate muscle regeneration, but at a different level. MSCs from bone-marrow expressed higher matrix-remodeling and proangiogenic factors implied in the retention, recruitment and migration of cells involved in vessel remodeling. In a contradictory study, Kim et al. (78) found that human ASCs had a higher therapeutic potential than human BM-MSCs in a similar model explained by a better proangiogenic action. The differences between these studies is the origin of the cells and also the isolation protocol. This highlights that in addition to tissue origin, isolations protocols could also influence the therapeutic properties of the cells. Indeed, cell biology, composition and viability can be disrupted by separation methods that vary greatly between production sites. In the case of scleroderma, Maria et al. (65) compared human ASCs and human MSCs in a murine model of diffuse SSc and underscored a significantly better effect with ASCs. They have especially found a higher secretion of matrix remodeling and anti-inflammatory factors by these latter, enabling improvement on skin thickness. Regarding fibrosis, both cells were able to reduce it in this preclinical model. These results indicate that tissue choice is important and depends on the pathology to treat and the cell functions of interest.

ADSCs are emerging as an alternative stem cell source for cell-based therapies as well as adipose-derived stromal vascular fraction containing ADSCs. A recent review classified a total of 41 papers describing the factors which modified ADSCs viability and function. These factors including age, gender, body mass index, donor site preference, diabetes mellitus or exposure to previous therapy, although this was not uniformly seen across all studies (79). All these factors, like in case of SSc patients, interact and future studies using ADSCs need to take them into consideration. Then, the same team characterized ADSCs from a cohort of six SS patients in comparison to six healthy age- and sex-matched controls. They indicated that the proliferation and migration capacity of ADSC issued from SS patients is reduced (68). In contrast, Capelli et al., showed that ADSCs from SSs patients had similar surface phenotype and multilineage differentiation capabilities. They did not observe any difference between ADSCs from SS patients and healthy donors neither in PBMC proliferation inhibition assays nor in ADSCs/Endothelial Cells cocultures. Moreover, this effect was enhanced under hypoxic conditions in all of the cocultures. They conclude that autologous ADSC grafting may represent a possible therapeutic option for SS (80). Finally, the previously described study of Maria et al. comparing the efficacy of xenogeneic BM-MSCs with ADSCs in a mouse model of HOCl-induced diffuse SS showed that ADSCs were significantly more efficient to reduce skin fibrosis than BM-MSCs (65). Other sources also emerged recently in clinical trials such as umbilical cord (UC-MSCs), in a recent trial, the association of plasmapheresis and allogeneic UC-MSCs transplantation led to a clinical benefit for the lung of SsS patients (73).

#### Medium and conditions of culture

Duration of expansion as well as culture conditions have a significant impacts on MSC proliferation, functional properties as well as on potential cytogenetic abnormalities. As a consequence, MSCs environment during expansion, including medium composition, needs to be thoroughly evaluated in the clinical process development and population doubling level (PDL) number must be limited to maintain maximal cell potential and safety *in vivo*.

Like isolation, no standardized expansion protocols yet exist between production facilities. MSCs seeding density can differ but it is now accepted that this criterion can have a significant impact on cell quality and yield. Our team noticed a significant difference in term of expansion rate between BM mononuclear cells seeded at 50,000 vs. 200,000/cm^2^. Marrow seeded at a lower concentration produces a better yield of MSCs, potentially due to a lesser concentration of red blood cells that can have a harmful effect on MSCs (data not published). Therefore, seeding concentration from primary tissue and also when passaging the cells need to be optimized early in the process development.

Presently, fetal bovine serum (FBS) is still widely used in MSCs productions. When associated with growth factors, in particular FGF-2, or cytokines such as PDGF-BB or EGF, it allows a great cell proliferation *in vitro*. With the combination of ascorbic acid, FGF-2 and PDGF-BB, MSCs are able to increase cell doublings when compared to each factor alone. However, surprisingly, some studies have shown that these factors could induce a loss of the differentiation potential of MSCs especially when cultured for a long time (81). Recent data suggest that the addition of heparan sulfate glycosaminoglycan in the media could promote selection of MSCs with increased self-renewal and with an enhanced survival *in vitro* and *in vivo*. In addition to lot to lot variability, FBS is derived from animals and represents a potential contamination risk. Consequently, the use of human platelet lysate (HPL) or serum-free medium (SFM) has been progressively preferred. In our lab, we demonstrated that, whatever the passage, there is a better proliferation of the cells when they are seeded in a medium containing 5–8% HPL in comparison with a medium containing 10% FBS and FGF-2. SFM is a chemically defined media that presents the advantage to allow a reproducibility between batches, but it also has drawbacks because of its high cost and the confidentiality of its composition. The choice of medium is very important and need to be tested to ensure that it doesn't negatively affect MSCs potential at least *in vitro*.

#### Priming

Many authors described the impact of SSc pathological context on MSCs, underlining their great sensitivity to their environment. However, it has been described that co-culture of SS-MSCs with endothelial cells from healthy donor could re-programs these cells toward a pro-angiogenic behavior (46). In this line, Fonteneau et al., showed that serum-mediated oxidative stress from SSc patients affects MSCs function. However, even if some functional properties of MSCs were affected upon culture with patient serum, MSCs can adapt to the oxidative environment and exert their therapeutic effect (69).

MSCs paracrine activity as well as exosomes release can be notably modulated by various extracellular signals form the microenvironment including soluble factors, gas, extra-cellular matrix and mechanical stimulation (82). Thereby, this again supports the idea that culture conditions need to be optimized to elicit a particular response and the expected therapeutic effect.

Among soluble signaling factors, TNF-alpha and IFN-gamma, for example, have been shown to induce secretion of CXCL9, CXCL10, IL-6, HGF, VEGF, and TGF-β by MSCs, which then become immunosuppressive by suppressing T cell proliferation. TGF-β is also able to induce MSCs secretion of multiple immunosuppressive factors. MSCs can also change their paracrine signaling in response to a serum-free medium or in response to gas modification. It was demonstrated that serum-starvation associated to 1% O_2_ culture induced the MSCs secretion of extravesicles (EVs) that contain more PDGF and EGF and that are able to induce angiogenesis via the NFkB pathway in a dose dependent manner (83). Hypoxia during culture might also provide pH and medium stabilization allowing a prevention of senescence, an improved genomic stability in BM-MSCs after genotoxic stress as well as the retention of their immunophenotype (84). Concerning effects of low oxygen on proliferation rate and differentiation potential, results are contradictory and seem to be linked to the applied percentage of O_2_. Indeed, Holzwarth et al. have in particular shown an impaired osteogenic differentiation at 1% O_2_ that was restored at 3% O_2_ (85). Physical micro-environment can influence MSCs as shown in 3D experiments developing spheroidal aggregates within which, cells shape, polarity and interactions are modified. MSCs in spheroids were shown to secrete high amount of PGE2 and to inhibit pro-inflammatory factors secretion by stimulated macrophages (86). At last, mechanical forces such as shear stress, tension and compression can modify MSC comportment and secretome.

#### Fresh vs. cryopreserved cells

The use of fresh cells is logistically difficult because it implies to harvest the required number of cells (dose/weight concentration) at the injection scheduled date and to wait few weeks for cell expansion but it remains entirely possible for many diseases including scleroderma. The interest of cryopreservation is the possibility to entirely evaluate the final product in terms of quality and safety prior to injection and to store and thaw MSC as needed with a stock available at any time. In addition, this allows the possibility to select the best donors (based on potency assays results *in vitro*).

Like for cell culture process, no standardized method is described for MSC cryopreservation. As suboptimal protocols can deeply cause cells damage and compromise their survival and stability, freezing protocol need to be optimized to retain the cells characteristics (87). To maximize cell recovery, cells should be harvested at the right time, that is to say in the exponential phase of growth and not at confluence, and cryopreservation medium composition needs to be optimized. It generally contains between 5 and 10% dimethyl sulfoxide (DMSO) solution but as it is potentially toxic, its replacement by sugars such as lactose, sucrose, trehalose, and raffinose is actually tested. As a base solution, balanced salt solutions as well as culture medium supplemented with FBS or, better, human albumin can be used. The freezing step should be done with a device applying a cooling rate of about 1°C per min, and cells viability and functionalities need to be checked post-thaw.

Many groups using diverse protocols for cryopreservation of MSCs from various origin have reported no negative effects on expansion, phenotype profile, differentiation and immunomodulating potential. As MSCs seem more robust than other cell types, this mode of conservation when optimized, can allow a good MSC survival, conservation of their characteristics and a lack of malignant transformation (88).

At contrary, some groups reported that cryopreservation could damage cells in particular by increasing necrosis and apoptosis and could impair immunosuppressive activities of MSC. Moll et al. showed that after a freezing step, thawed MSCs exhibit increased triggering of Instant Blood Mediated Inflammatory Reaction (IBMIR) as well as activation of the complement cascades leading to a lysis of the cells when exposed to normal human serum. They also demonstrated reduced immunomodulatory properties *in vitro* with a worse suppression of PHA-stimulated MLRs and an impaired efficacy in GVHD clinical trials (89).

#### MSCs production: new approaches

Conventional cell culture flasks or factories can be used to assure MSCs expansion for a single patient in the case of hospital exemption (autologous or allogeneic cells). For allogeneic MSCs production and banking, on the other hand, hyperstacks and especially bioreactors begin to have significant interest since they allow expansion of cells at a large scale in a closed environment. These devices can monitor temperature, dissolved oxygen, pH level and agitation, and allow a reproducible and more standardized process (87). Different types of bioreactors compatibles with GMP manufacturing are actually commercialized, proposing various technologies depending on the type of cells and the desired final product (i.e cells or conditioned medium). For MSCs, dynamic suspension culture using microcarrier beads in a stirred-tank bioreactor is often used.

As described previously MSC could exert their therapeutic effect through the secretion of a large panel of bioactive molecules but also by the transfer of extracellular vesicles (EVs) containing proteins, RNA or MicroRNAs (miRNAs). miRNAs are small non-coding RNAs that function as post-transcriptional regulators of gene expression (90). A study of Chen et al. showed that MSCs could transfer miR-151-5p to the recipient BM-MSCs in SS mice. They also showed that the delivery of miR-151-5p could rescue osteopenia, impaired bone marrow MSCs, tight skin, and immune disorders in SS mice (66). Therefore, EVs/miRNA transfer may play a significant role in the MSCs therapies.

#### Safety controls

To ensure patient safety, cytogenetic tests have to be considered when using MSCs in therapeutics. The conventional test used in clinical production is a Giemsa-banding (G-banding) karyotype analysis. ISCT recommends not to inject cells if at least 2 out of 20 metaphases have an identical abnormality—a recommendation based on the 2009 edition of the International System for Human Cytogenetics Nomenclature (91). However, this test has its limits and does not necessarily warrant a high standard of quality required in clinical use of MSCs. One of the limits of G-banding karyotype analysis is that it considers mitotic cells that have been arrested in the metaphase portion of the cell cycle, which accounts for <0.1% of tested cells with the majority being in interphase. In addition, this test identifies only abnormalities greater than 5-10 mega-bases, whereas structural abnormalities such as inversions, deletions and duplications within the same chromosome are not detected. The result of this test is therefore far from being a representation of the overall cell population obtained after culture. Importantly, if G-banding karyotype analysis is positive, it is not possible to make a connection with a selective proliferation advantage and a transformation of the cells with abnormalities. Indeed, MSCs can acquire random and spontaneous genetic aberrations during their *ex vivo* expansion, mainly aneuploidies, but these chromosomal alterations are non-recurrent and interpreted as being related to senescence (92, 93). Based on current literature, no evidence exists suggesting that MSCs with karyotypic abnormalities at the time of injection are deleterious to the patient. When MSCs from bone marrow presenting aneuploidy were injected into immunodeficiency mice, they did not produce tumors after 8 weeks (94). In addition, injected MCS have a limited lifespan, don't persist *in vivo* and their transformation, a very rare event, has never been reported in humans after almost 20 years of clinical use. It would appear that, unlike other species, human MSCs exhibit resistance to spontaneous transformation (95). Investigations conducted following the occurrence of karyotypic abnormalities (aneuploidy) in MSC cultures, found that there are no elements to promote a risk of transformation: no selective clone advantage, complete proliferation arrest between 35 and 52 population doublings, no expression of telomerase (hTERT) and, interestingly, no modification of expression of genes involved in transformation (p53 and p21) was observed and a decrease of c-myc during culture. These results indicate no sign in favor of genetic transformation or a potential tumorigenicity (94). Another team (96), for its part, has shown that human MSCs are genetically stable until passage 4 (increased incidence of abnormalities during later passages) and that they can be cultured for long-term *in vitro* without losing their immunomodulatory capacity or multipotency, even when they had karyotypic abnormalities.

Finally, techniques that are more sensitive than karyotype have shown large number of cellular mosaics (somatic mutations) accumulated in normal adult tissues, particularly in elderly individual (97), highlighting the complexity of genetic analysis interpretations. The hTERT assay for telomerase expression seems therefore more appropriate for the quality control of MSCs from a safety point of view, which is more predictive of a risk of tumorigenicity of the cells. Indeed, activation of the enzyme telomerase is considered one of the classic markers of cancer, since 90% of cancer cells and 70% of human tumor lines have overexpression of telomerase and thus replicative immortality.

To ensure safety and minimize the risk of cell transformation, a cytogenetic assay should be performed in addition to telomerase expression studies, which would ideally consist of G-banding karyotyping in addition to more sensitive spectral karyotyping (SKY) or CGH array designed to detect abnormalities ≤50kb (91).

#### Stability and route of administration

MSCs final product “expiration” is often defined as being between 4 and 6 h at 4°C. But some stability data suggest that conditioned MSCs demonstrate a loss of clonogenicity and anti-inflammatory potential *in vitro* after 4 h, even if viability remains good. To enhance cell stability, the final product can be stored at 4°C in a specialized hypothermic storage media such as HypoThermosol prior to injection even if this solution seems to pause the cell metabolism and that a recovery delay after rewarming would be appreciated (98). If freeze-thawed MSCs are used, a period of cell recovery post-thaw would allow optimize cell function. Indeed, while freshly thawed MSCs have showed significantly reduced viability as well as a decreased ability to suppress T-cell proliferation *in vitro* compared to actively growing MSCs, thawed MSCs that were cultured for 24 h regained these immunomodulatory–linked functions (98).

The MSC delivery is also an important criterion to discuss before a clinical use. MSCs can be delivered systemically even though it has been suggested that the majority of cells are trapped by the lungs after infusion and are eliminated thereafter. Consequently, the choice of the site of injection will influence the cell fate and capacity to migrate to injured tissues. However, Maria et al. (65) described a therapeutic effect of MSCs on the skin of SSc mice while no injected cells were found in this organ (half being found in the lung after 48 h and total clearance being observed after 7 days) suggesting that the observed effect couldn't be associated with the MSCs migration to the injured skin.

Locally MSCs injections into tissues (intramuscular or other way) are also used in clinic and provide promising results. Since MSCs are fragile and tend to be removed rapidly from the recipient's body, some groups are working on the encapsulation of MSCs to allow a higher cell protection and a slow delivery of the therapeutic product (99).

### Perspectives

MSC-based therapy represents a potential hope for the patients inflicted by an advanced form of SSc. MSCs require a high level of competence and extreme care which should be taken at each stage from cell isolation to clinical trial evaluation. Indubitably, the understanding of MSCs biology has grown profoundly over the last decade. The ability of MSCs to positively influence processes such as immunosuppression, angiogenesis and inflammation generated a lot of interest and enthusiasm from clinicians and researchers alike. However, fundamental questions related to their biological properties especially when it comes to their mode of action *in vivo* are yet to be elucidated. Development of novel highly efficient *in vitro* functional tests and potency assays are a prerequisite to clinically effective and reproducible treatment. Moreover, clinical use of MSCs warrants stringent testing, which is already imposed by legislations and relevant regulatory authorities. An ideal test would assist in selection of cells of the highest quality, which will in turn boost the therapeutic potential and efficacy of cell therapy *in vivo*. EMA, in their 2011 guidelines, recommend a semi-quantitative functional assay such as cellular migration, immunosuppression which may involve phenotyping and profile studies in the form of membrane marker expression or cytokine synthesis. Although some tests have shown efficacy both *in vitro* and *in vivo* (100), systematic correlation between results obtained *in vitro* and *in vivo* are still lacking. It is apparent that many questions remain unanswered, however what is becoming clear is that MSCs-based therapy should considered as a safe and potentially efficient therapeutic option in the management of advanced stage of SSc.

## Author contributions

CM wrote the manuscript with input from all authors. All authors (JP, MA, NF, EB, and J-JL) provided critical feedback and helped shape the manuscript.

### Conflict of interest statement

The authors declare that the research was conducted in the absence of any commercial or financial relationships that could be construed as a potential conflict of interest.
